# A Controlled Release Codelivery System of MSCs Encapsulated in Dextran/Gelatin Hydrogel with TGF-*β*3-Loaded Nanoparticles for Nucleus Pulposus Regeneration

**DOI:** 10.1155/2016/9042019

**Published:** 2016-09-27

**Authors:** Yibo Gan, Sukai Li, Pei Li, Yuan Xu, Liyuan Wang, Chen Zhao, Bin Ouyang, Bing Tu, Chengmin Zhang, Lei Luo, Xiangdong Luo, Xiumei Mo, Qiang Zhou

**Affiliations:** ^1^National & Regional United Engineering Laboratory of Tissue Engineering, Department of Orthopedics, Southwest Hospital, Third Military Medical University, 30 No. Gao Tan Yan Street, Shapingba District, Chongqing 400038, China; ^2^Department of Orthopaedics, Xinqiao Hospital, Third Military Medical University, Chongqing 400038, China; ^3^Institution of Burn Research, Southwest Hospital, Third Military Medical University, Chongqing 400038, China; ^4^College of Chemistry and Chemical Engineering and Biological Engineering, Donghua University, Shanghai 201620, China

## Abstract

Mesenchymal stem cell- (MSC-) based therapy is regarded as a potential tissue engineering strategy to achieve nucleus pulposus (NP) regeneration for the treatment of intervertebral disc degeneration (IDD). However, it is still a challenge to induce MSC differentiation in NP-like cells when MSCs are implanted into the NP. The purpose of this study was to construct poly(D,L-lactide-co-glycolide) (PLGA) nanoparticles as carriers for TGF-*β*3 controlled release and establish a codelivery system of a dextran/gelatin hydrogel with the nanoparticles for long-term processing of discogenesis differentiation. TGF-*β*3-loaded PLGA nanoparticles were prepared by the double-emulsion solvent evaporation method and seeded uniformly into the hydrogel. Morphological observations, an assessment of the release kinetics of TGF-*β*3, a cytotoxic assay, a cell proliferation test, a biochemical content assay, qRT-PCR, and immunohistological analyses of the codelivery system were conducted in the study. The results showed that the TGF-*β*3-loaded nanoparticles could release TGF-*β*3 gradually. The codelivery system exhibited favorable cytocompatibility, and the TGF-*β*3 that was released could induce MSCs to NP-like cells while promoting ECM-related biosynthesis. These results suggest this codelivery system may be employed as a promising carrier for discogenesis of MSCs* in situ*.

## 1. Introduction

Intervertebral disc degeneration (IDD) is a common disease that causes lower back pain, neck pain, and even disabilities [1]. IDD affects patients' quality of life and leads to a large economic burden. Currently, the common treatments of IDD consist of bed rest, exercise, physical therapy, and surgery [2]. However, all of these treatments only relieve the symptoms without targeting the etiology of IDD. Thus, there is no approved therapy for IDD, and there is an urgent need for treatments that target the etiology of IDD to repair the intervertebral disc (IVD).

The IVD is a complex joint that consists of three parts, the central hydrated nucleus pulposus (NP), the outer lamellar annulus fibrosus (AF), and the cartilage endplates (CEP), connecting the adjacent vertebras. IDD is believed to originate in NP, and improper load on IVD leads to NP dehydration and subsequently weakens IVD's capacity to absorb compression [3]. Thus, rehydration of the degenerative NP may be the key to treating IDD. Previous studies have indicated that the bioactivity and amounts of NP cells (NPCs) are decreased significantly in IDD, resulting in a lower secretion of extracellular matrix- (ECM-) related proteins, including aggrecan and type II collagen [4]. Specifically, the main function of aggrecan is to absorb water molecules into the NP, contributing to the compression absorption function of the IVD. Therefore, increasing NPC quantity and bioactivity may promote the restoration of NP hydration. Due to the poor self-regeneration capability of NPCs, cell-based therapy could be a promising strategy for the treatment of IDD [5]. Of all of the cell-based approaches, implantation of mesenchymal stem cells (MSCs) encapsulated in hydrogels has received the greatest attention [6].

An advantage of the MSC-based strategy is that autologous cells are easily harvested and expanded rapidly* in vitro*. There is greater potential to regenerate the NP with larger amounts of implanted cells. Another advantage is that MSCs are able to differentiate to an NP-like phenotype with some proper biochemical directions, such as transforming growth factor *β* (TGF-*β*) [7] and differentiation factor 5 (GDF5) [8]. The TGF-*β* family is one of the exogenous growth factors widely used to induce MSCs to discogenesis differentiation. Generally, high-density MSCs in a three-dimensional (3D) carrier were cultured with a serum-free induction medium with TGF-*β* isoforms* in vitro* for chondrogenic differentiation, especially with TGF-*β*3 [9], and, subsequently, the hybrid was implanted for NP regeneration [10]. Hydrogels are considered proper carriers because of their similar rheological property to the NP and favorable cytocompatibility [11]. However, this strategy is difficult to apply for the treatment of early stage IDD. Due to the structural integrity of AF in early stage IDD, IVD fenestration should be made before implanting a bulk cell-hydrogel hybrid, which could possibly accelerate IDD [12]. Therefore, a novel alternative may involve using an injectable hydrogel as a carrier of MSCs. The injectable hydrogels are in the liquid state before implantation and harden after injection* in vivo*. The process allows minimal invasion through the AF. It is beneficial for avoiding extrusion after implantation while adapting to the irregular shape of the defect in the NP cavity [13]. The precursor solution can also be combined with growth factors and cells to reverse disc degeneration [14]. However, it is still a challenge to maintain long-term MSC differentiation* in situ* [15]. The half-life of TGF-*β*3 is only tens of minutes* in vivo* and it can be easily diluted in body fluid diffusion. If growth factors were combined directly with the hydrogel, they would soon be degraded or eliminated. Therefore, it is difficult to affect TGF-*β*3 by physical mixing [15]. Thus, it is particularly important to construct a suitable delivery system for the sustained release of growth factors.

The rapid development of the FDA-approved poly(D,L-lactide-co-glycolide) (PLGA) may be useful for long-term differentiation [16]. PLGA is a copolymer of PLA and PGA with favorable biocompatibility, favorable biodegradability, and low immunogenicity. Recently, PLGA was applied to drug delivery and tissue engineering applications among the most attractive polymeric candidates [17]. The preparation of nanosized particles has increased with advances in traditional methods because nanoparticles could be delivered to various body parts with a wide range of drugs for sustained release [18]. PLGA nanoparticles (PLGANPs) can be targeted to a specific tissue and release the drug locally by endocytosis [19]. Such PLGANPs have been widely used to develop anticancer drug delivery system, such as paclitaxel, doxorubicin, 5-fluorouracil, and dexamethasone, which were effectively formulated via PLGANPs [20]. It has also been incorporated in tissue engineering scaffold for control over spatial and temporal release of growth factors. For instance, glial cell-line derived neurotrophic factor (GDNF) was encapsulated in PLGANPs when implanted into the spinal cord to induce an increase in neuronal survival reported by Wang et al. [21]. If PLGANPs are used as carriers for a gradual release of growth factors into the IVD, local maintenance may lead to sufficient concentrations of TGF-*β*3 in the long-term process of NP regeneration. The injectable hydrogel coupled with the sustained release nanoparticles could meet the clinical needs of an ideal MSC-based minimally invasive implantation.

In this study, TGF-*β*3-loaded PLGANPs were prepared and seeded into a dextran/gelatin hydrogel to construct a novel codelivery system for inducing long-term MSC differentiation* in situ*. The overall objective of this study was to investigate the potential of the codelivery system for NP regeneration. To achieve this, we first evaluated the release kinetics of the TGF-*β*3 and confirmed that the TGF-*β*3-loaded PLGANPs could release enough TGF-*β*3 within an appropriate discogenesis time frame. Second, we investigated the cytocompatibility of the TGF-*β*3-loaded PLGANPs. Finally, we investigated whether the codelivery system could support cell proliferation, discogenesis differentiation, and functional biosynthesis of MSCs.

## 2. Materials and Methods

### 2.1. Preparation of the TGF-*β*3-PLGANPs

The TGF-*β*3-loaded PLGANPs were prepared using the double-emulsion solvent evaporation method (W/O/W) as previously described [22]. In order to reach the concentration of TGF-*β*3 as previously recommended for the discogenesis differentiation of MSCs, the target release amount of the TGF-*β*3 was 10 ng in 1 mL culture medium [23]. Briefly, 10 *μ*g of the TGF-*β*3 (PeproTech, USA) was dissolved in 0.1 mL of distilled water (W_1_). Subsequently, the PLGA 50 : 50 (200 mg, 24–38 kD, Sigma-Aldrich, USA) was dissolved in 1 mL dichloromethane (O, Sigma-Aldrich). Later, the TGF-*β*3 solution was added dropwise to the PLGA solution. Then the solution was treated ultrasonically in an ice bath. This primary emulsion was added dropwise to 6 mL of 1% polyvinyl alcohol (PVA, Sigma-Aldrich, USA) aqueous solution (W_2_) and treated ultrasonically again. The obtained W_1_/O/W_2_ emulsion was transferred into 20 mL of the 0.3% PVA aqueous solution and stirred for 4 hours with a propeller stirrer. Subsequently, the solid particles were collected after centrifugation. Finally, the obtained precipitates were washed for three times with deionized water. The nanoparticles were harvested by lyophilization and stored at 20°C in desiccative condition.

### 2.2. Morphological Observations

The morphology of the TGF-*β*3-loaded PLGANPs was examined by the scanning electron microscopy (SEM, S3400N II, Hitachi, Japan) and the transmission electron microscope (TEM, TECNAI 10, Philips, USA). Briefly, 20 *μ*L of the PLGANPs suspension was dropped on the mica, followed by being air-dried overnight. Then morphology of the TGF-*β*3-loaded PLGANPs was examined by the SEM after spraying. For the TEM examination, the PLGANPs suspension was dropped on the copper mesh with the film. Then the excess liquid on copper mesh edge was absorbed with filter paper. Next, drop phosphotungstic acid negative stain solution for 2 minutes, followed by gently washing for three times. The nanoparticles were examined by the TEM after air drying. The particle size was determined by laser particle size analyzer (Zetasizer V7.02, Malvern, UK) (*n* = 3).

### 2.3. Encapsulation Efficiency (EE)

The EE in the PLGANPs was estimated by a “two-step” extraction method [24]. Briefly, about 5 mg of the lyophilized TGF-*β*3-PLGA nanoparticles was added to 1 mL of acetonitrile. The supernatant was discarded and the precipitation was pelleted down after centrifugation. The pellet was dissolved in the PBS and the samples (in duplicate) were centrifuged again. The supernatant was preserved after centrifugation and its residue was dissolved in NaOH. The protein amount in aqueous solution and NaOH (the actual theoretical loading amount of TGF-*β*3 encapsulated in the 5 mg of PLGANPs, *M*_actual_) was determined by the ELISA kit (Thermo Fisher Scientific, USA) (*n* = 3). The EE of the TGF-*β*3 in the PLGANPs was calculated as follows:

(1) $$\begin{array}{c} EE=\frac{M_{actual}}{M_{theoretical}}\times 100\%, \end{array}$$

where *M*_theoretical_ is the theoretical loading amount of the TGF-*β*3 encapsulated in 5 mg PLGANPs.

### 2.4. Release Kinetics of the TGF-*β*3

The release kinetics of the TGF-*β*3 were determined* in vitro* as previously described [25]. Briefly, the TGF-*β*3-loaded nanoparticles (50 mg) were suspended with 3 mL of the PBS, shaking at 100 rpm at 37°C. The supernatant was collected and stored at −20°C on the predetermined time points (days 1, 3, 7, 10, 14, 21, and 28) after centrifugation. Then, an equal amount of the fresh PBS was added and incubated as previously described. The released quantity of the TGF-*β*3 was determined by the ELISA kit. The nanoparticles' release kinetic curve was obtained using the accumulated release percentage (*n* = 3).

### 2.5. Isolation and Culture of Mouse MSCs

The usage of the animal followed the guidelines of Local Animal Ethics Committee (SYXK (YU) 2012-0012). Bone marrow-derived MSCs were collected from 6-week-old Balb/c mouse as in a previous study [26]. Briefly, the mouse was killed by cervical dislocation. Bone marrow was harvested by flushing the femurs and tibiae with the complete culture medium [Dulbecco's modified Eagle's medium (DMEM, HyClone, USA) supplemented with 10% fetal bovine serum (FBS, Gibco, USA) and 1% penicillin/streptomycin (Gibco, USA)]. MSCs were isolated from marrow cells followed by density-gradient centrifugation (1.077 g/cm^3^). Remove the nonadherent cells after 3 days and collect the adherent cell by trypsinization (0.05% trypsin-EDTA, Gibco, USA) when reaching 90% confluence. The medium was changed every 2 days.

### 2.6. Live/Dead Assay

Four groups were chosen for the cytotoxicity assay: (1) TGF-*β*3-PLGANPs: supplemented with the TGF-*β*3-loaded PLGANPs at the concentration of 1000 *μ*g/mL in the culture medium; (2) TGF-*β*3 group: supplemented with the TGF-*β*3 at the concentration of 10 ng/mL in the culture medium; (3) PLGANPs group: supplemented with the blank PLGANPs at the concentration of 1000 *μ*g/mL in the culture medium; (4) control group: no supplement in the culture medium. Except for the TGF-*β*3 group, the MSCs or MSCs-seeded hybrids were cultured with the complete culture medium. Cells cultured with 0.1% Triton (Beyotime Biotechnology, China) were chosen as negative control. In order to evaluate the cytotoxicity of the PLGANPs, MSCs were seeded on the 24-well plates at the density of 1 × 10^4^/well. After 1 day, the culture medium was removed and 1 mL culture medium containing the PLGANPs at the concentration of 1000 *μ*g/mL was added to the wells after the cell attachment. The cytotoxicity was evaluated after 7 days' coculture* in vitro* by a LIVE/DEAD Viability/Cytotoxicity Assay Kit (Invitrogen, USA) according to the manufacturer's instructions. The stained cells were observed with a fluorescent microscope (LSM 510, Zeiss, Germany). Living cells percentage was calculated by Image J software (Wayne Rasband, National Institute of Health, USA). Three images from three samples were evaluated for each group.

### 2.7. Establishment of the Codelivery System

**Figure 1:**
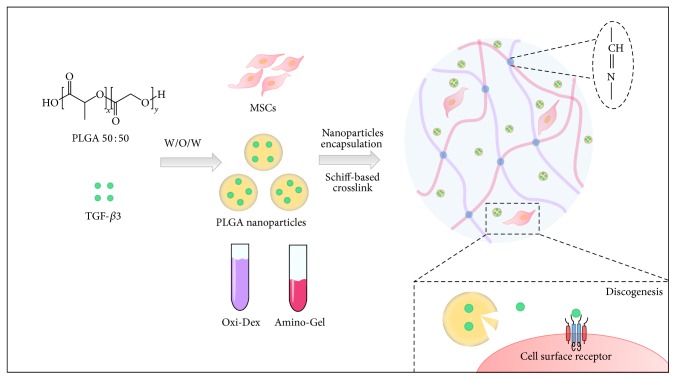
Schematic diagram of establishing the codelivery system of MSCs encapsulated in the dextran/gelatin hydrogel with the TGF-*β*3-loaded nanoparticles. After the TGF-*β*3-loaded PLGANPs were prepared using the W/O/W method, the MSCs and the TGF-*β*3-loaded PLGANPs were incubated with the Oxi-Dex and amino-Gel solution. The codelivery system was formed by the Schiff-based reaction between the Oxi-Dex and amino-Gel. The TGF-*β*3 could be released gradually with degradation of the PLGANPs for long-term discogenesis differentiation.

The dextran and gelatin were obtained from Sigma-Aldrich. The oxidative dextran (Oxi-Dex) and the amino gelatin (amino-Gel) were prepared as our previous study [27]. The resulting solution was dialyzed (MWCO 7000, JinKeHongDa, China) against distilled water for 3 days. Then, it was lyophilized to obtain the products. Then the dextran and the gelatin were dissolved in the PBS to reach the concentration of 20%. The encapsulation and the sustained release process were exhibited in Figure 1. Briefly, the TGF-*β*3-loaded PLGANPs or the blank PLGANPs were suspended by the Oxi-Dex solution. Next, the amino-Gel solution was added to the mixture at a mass ratio of dextran versus gelatin to 3 : 5 at the concentration of 20%. The final solution was transferred to a mold rapidly for gelation for 5 min. The density of PLGANPs in the hydrogel was 1000 *μ*g/mL, which was set to reach the TGF-*β*3 concentration of 10 ng/mL in the system according to the release kinetics. 1 mL of culture medium was added and changed every day for the following tests. For the cells encapsulation, the MSCs (P3–P5) were premixed with the prepolymer solutions at a density of 5 × 10^6^ cells·mL^−1^. And the hybrid was shaped to a cylinder (Φ = 10 mm, height = 6 mm) and cultured in our bioreactor with a circulating system. The hybrids were divided into four groups: (1) TGF-*β*3-PLGANPs: the MSCs encapsulated in the hydrogel with the TGF-*β*3-loaded PLGANPs at the density of 1000 *μ*g/mL, cultured with the complete medium; (2) TGF-*β*3 group: the MSCs encapsulated in the hydrogel with no supplement, cultured with the medium containing TGF-*β*3 at the concentration of 10 ng/mL; (3) PLGANPs group: the MSCs encapsulated in the hydrogel with the blank PLGANPs at the density of 1000 *μ*g/mL, cultured with the complete medium; (4) control group: the MSCs encapsulated in the hydrogel with no supplement, cultured with the complete medium.

### 2.8. Cell Proliferation in 3D

To detect the cell proliferation of MSCs in the codelivery system, the hydrogels were transferred to a 24-well plate at the time point of days 7, 14, and 28. Then, fresh medium containing 10% CCK-8 was added for 3 h incubation. The absorbance of incubation solution was measured at 450 nm using a microplate reader (Model 550, Bio-Rad, USA) (*n* = 4).

### 2.9. Biochemistry Assays

For testing the cell viability and ECM deposits, the MSCs-seeded hybrids were lyophilized at days 7, 14, and 28. DNA and glycosaminoglycan (GAG) content were analyzed as described before [28]. The hydroxyproline (HYP) content was quantified according to a previous method [29] (*n* = 3).

### 2.10. Real-Time PCR Assay

After 28 days of postseeding, cell-seeded hybrids were treated with TRIzol (Geno Technology Inc., USA). RNA was extracted according to the manufacturer's instruction. cDNA was generated by applying cDNA reverse transcription kit (Life Technologies, USA) and diluted to 5 ng/*μ*L. Gene expression was analyzed by quantitative real-time PCR (Applied Biosystems 7500, Thermo Fisher Scientific, USA). Data were calculated by the 2^−ΔΔCt^ method. The primers used in this study are shown in Table 1.

### 2.11. Immunohistochemical Analysis

For histological analysis of cell morphology and ECM secretion, hybrids from each time point (7, 14, and 28 days) were moved into paraffin. Then in order to visualize the ECM deposition of the hybrids, the sections were immunostained by collagen II and aggrecan as in reported method [30]. The aggrecan (1 : 400, sc-16492, Santa Cruz Biotechnology, USA), type II collagen (1 : 400, ab34712, Abcam, UK), and goat anti-mouse horseradish peroxidase-conjugated secondary antibody (1 : 1000, CW0102, Cwbiotech, USA) were applied in the analysis.

### 2.12. Statistical Analysis

The mean ± standard deviation (SD) was applied to present the quantitative data in this study. The differences between the groups were analyzed by ANOVA with SPSS (version 15.0, IBM, USA). Student-Newman-Keuls test was adopted to compare pairwise between the groups obtained. Differences were accepted as significant when two-sided probabilities were less than 0.05.

## 3. Results

### 3.1. Morphology and Release Kinetics of TGF-*β*3-Loaded PLGANPs* In Vitro*

**Figure 2:**
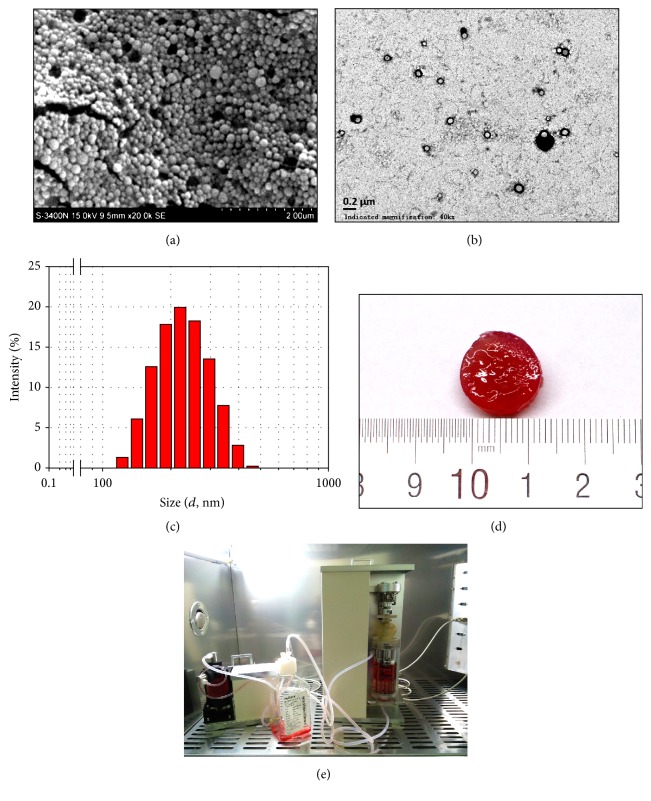
(a) Scanning electron microscopy micrographs of the TGF-*β*3-loaded PLGANPs (magnification: 50,000x, scale = 2 *μ*m); (b) transmission electron microscopy of the TGF-*β*3-loaded PLGANPs (magnification: 500,000x, scale = 0.2 *μ*m), showing good dispersibility of the PLGANPs; (c) size distributions of the TGF-*β*3-loaded PLGANPs; (d) general views of the codelivery system of the MSCs encapsulated dextran/gelatin hydrogel with the TGF-*β*3-loaded PLGANPs after 28 days'* in vitro* culture; (e) a custom-made bioreactor system, including integrated servomotor and circulating system, for promoting TE-NP tissue formation.

The SEM of the TGF-*β*3-loaded PLGANPs is shown in Figure 2(a). The particles exhibited the smooth surfaces of the nanosize sphere. As shown in Figure 2(b), the TEM image showed that nanoparticles were dispersed well and that their size was uniform. The particle size of the tested sample presented a normal distribution (Figure 2(c)), and the diameter of the nanoparticles was 218.36 ± 2.76 nm. The gross morphology of the dextran/gelatin hydrogel with TGF-*β*3-loaded PLGANPs is shown in Figure 2(d).

**Figure 3:**
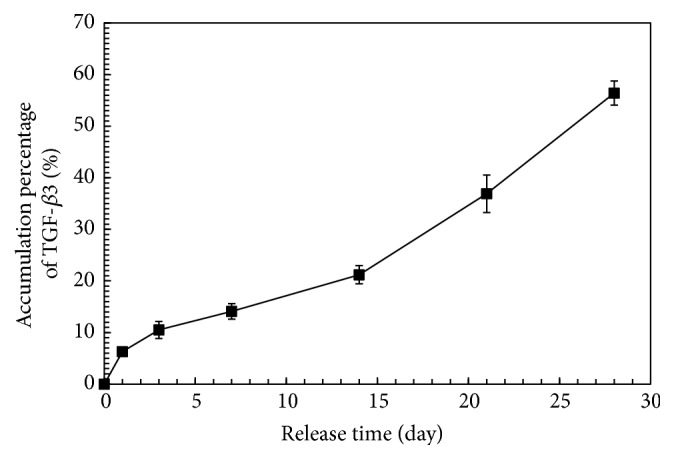
Release kinetics of the TGF-b3 from the PLGANPs. Data were expressed as means ± SD (*n* = 3).

The release kinetics of TGF-*β*3 are shown in Figure 3. On day 1, the TGF-*β*3 was released in a burst with a release percentage of 6.3 ± 0.9%. Subsequently, the sustained release percentage of the drug decreased and stabilized gradually during the middle stage of release from day 2 to day 14, and 21.2 ± 0.8% of the TGF-*β*3 was released from the nanoparticles at this stage. After 28 days, the cumulative release percentage reached 56.4 ± 2.4%. The EE of the TGF-*β*3 in the PLGANPs was 64.1 ± 2.57%. It indicated that the TGF-*β*3 loaded in the PLGANPs could be released stably for the long term.

### 3.2. Cytotoxicity of PLGANPs and Cell Proliferation of MSCs in the Codelivery System in Long-Term Culture

**Figure 4:**
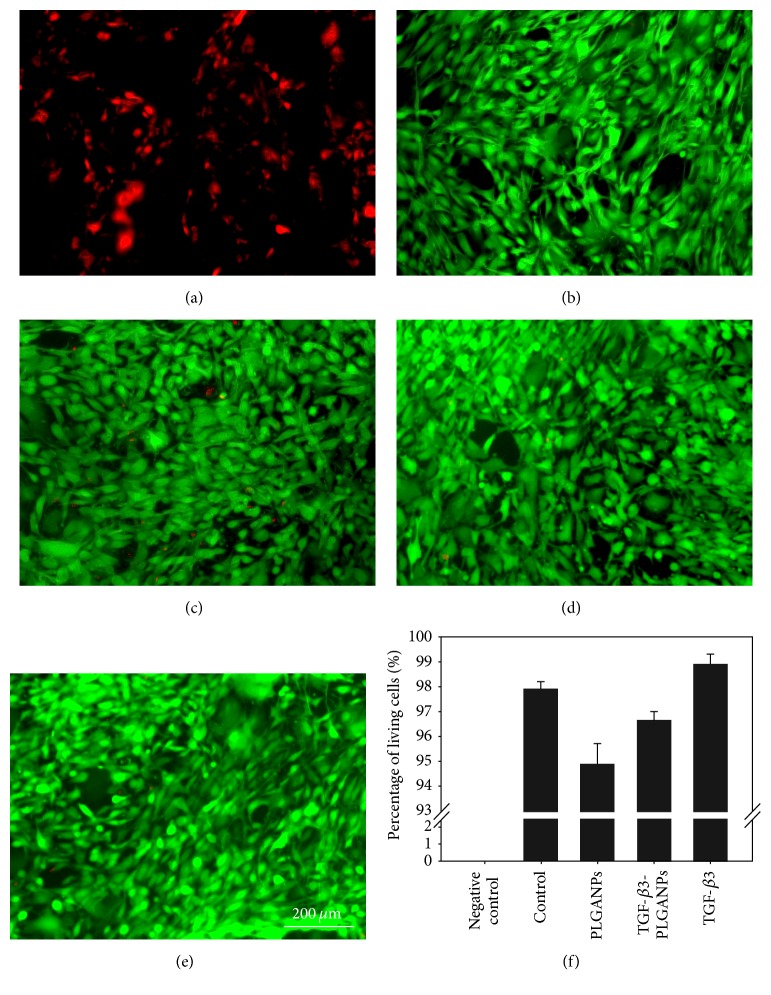
Cytotoxicity assay of the MSCs cocultured with (a) 0.1% Triton-X (negative control), complete culture medium containing; (b) no supplement (control); (c) 1000 *μ*g/mL blank PLGANPs (PLGANPs); (d) 1000 *μ*g/mL TGF-*β*3-loaded PLGANPs (TGF-*β*3-PLGANPs); and (e) 10 ng/mL TGF-*β*3 (TGF-*β*3) in the medium (magnification: 100x, scale = 200 *μ*m). Data were expressed as means ± SD (*n* = 3).

A fluorescent live/dead stain was used to assess the effects of PLGANP internalization. Living cells are stained by calcium AM, which yields a green fluorescence. Membranes of dead cells comprise EthD-1, yielding a red fluorescence. Fluorescent images of MSCs obtained 7 days after PLGANP exposure are shown in Figure 4. There is no significant difference in MSC viability among all of the groups on day 7 (*p* > 0.05), although almost all the dead cells were found in the group incubated with PLGANPs. The results demonstrated that PLGANPs had little cytotoxicity in the exposed concentration and good cytocompatibility when PLGANPs were loaded with the TGF-*β*3.

**Figure 5:**
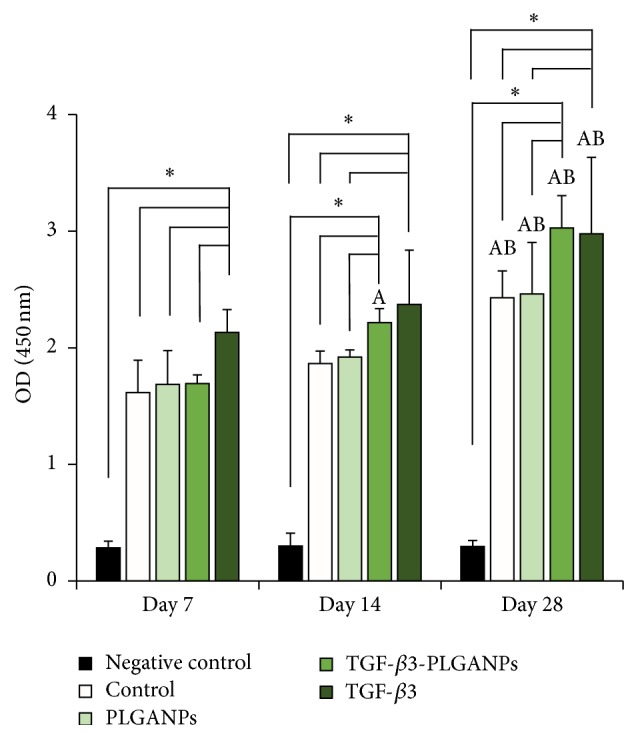
CCK-8 result of the MSCs cultured in the dextran/gelatin hydrogel encapsulated with no supplement (control), 1000 *μ*g/mL blank PLGANPs (PLGANPs), 1000 *μ*g/mL TGF-*β*3-loaded PLGANPs (TGF-*β*3-PLGANPs), and the hydrogels with no supplements cultured in the medium containing 10 ng/mL TGF-*β*3 (TGF-*β*3) for 7, 14, and 28 days. The hydrogels with no seeded cell were chosen as the negative control. The data were expressed as the mean ± SD (*n* = 4). The samples indicated with (*∗*) had a significant difference between the two groups (*p* < 0.05). The samples indicated with (A) had a significant difference compared to the same group on day 7 (*p* < 0.05) and (B) had a significant difference compared to the same group on day 14 (*p* < 0.05).

The CCK-8 assay was conducted to quantify the proliferation activity of MSCs within the codelivery system. The higher optical density (OD) indicated greater cell numbers. As shown in Figure 5, the cell proliferation activity of MSCs of the TGF-*β*3 group was statistically higher than all of the other groups after 7 days of postseeding (*p* > 0.05). On day 14, the MSCs supplied with TGF-*β*3 in medium or with TGF-*β*3-loaded PLGANPs exhibited a higher OD value than that of the other groups (*p* < 0.05). The cell metabolic activity was higher in the TGF-*β*3 group than in the TGF-*β*3-PLGANPs group, but the difference was not significant (1.1-fold, *p* > 0.05). After 28 days of postseeding, the medium containing TGF-*β*3 and the TGF-*β*3-loaded PLGANPs could promote significantly greater cell proliferation than the other groups (*p* < 0.05), but there was no obvious difference that could be found between the two groups (*p* > 0.05). The results indicate that TGF-*β*3-loaded PLGANPs could release active TGF-*β*3 in the codelivery system, and TGF-*β*3 could promote long-term cell metabolism.

### 3.3. Effect of the Codelivery System on MSC Phenotype

**Figure 6:**
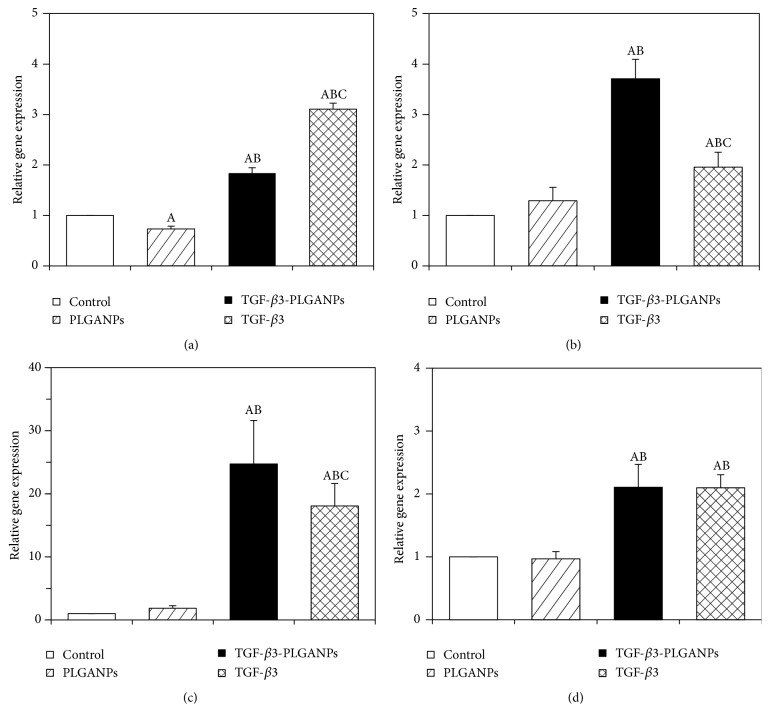
The expressions of (a) KRT18, (b) Shh, (c) aggrecan, and (d) type II collagen *α*1 of MSCs in the dextran/gelatin hydrogel with no supplement (control), 1000 *μ*g/mL blank PLGANPs (PLGANPs), 1000 *μ*g/mL TGF-*β*3-loaded PLGANPs (TGF-*β*3-PLGANPs), and the hydrogels with no supplements cultured in the medium containing 10 ng/mL TGF-*β*3 (TGF-*β*3) for 28 days. The expression levels were quantified using real-time PCR and normalized to the housekeeping gene, GAPDH. The data were expressed as the mean ± SD (*n* = 3). The samples indicated with (A) had a significant difference compared to the control group (*p* < 0.05), (B) had a significant difference compared to the TGF-*β*3-PLGANPs group (*p* < 0.05), and (C) had a significant difference compared to TGF-*β*3 group (*p* < 0.05).

The discogenesis phenotype of seeded MSCs on day 28 was investigated by qRT-PCR, as shown in Figure 6. In general, the TGF-*β*3 could induce MSCs to differentiate into NP-like cells. After 28 days of postseeding, the expression of KRT18 was upregulated significantly more in the TGF-*β*3 and TGF-*β*3-PLGANP groups than in the other groups (*p* < 0.05). In the TGF-*β*3 group, the expression was significantly elevated relative to the TGF-*β*3-PLGANP group (1.84-fold, *p* < 0.05). For constructs cultured in TGF-*β*3-loaded PLGANPs, the expression of Shh was 3.7-, 2.8-, and 1.9-fold greater than the control, PLGANP, and TGF-*β*3 groups, respectively (*p* < 0.05). Aggrecan expression in the TGF-*β*3-PLGANPs group was 24.7-, 13.3-, and 1.4-fold greater after 28 days of culture than the control, PLGANP, and TGF-*β*3 groups, respectively (*p* < 0.05). The expression of type II collagen *α*1 in the TGF-*β*3-PLGANP group was 2.1- and 2.2-fold higher than the control and PLGANP groups, respectively (*p* < 0.05), after 28 days of culture; however, there was no significant difference between the TGF-*β*3-PLGANP and TGF-*β*3 groups (*p* > 0.05). The phenomenon demonstrated that TGF-*β*3-loaded PLGANPs could induce differentiation toward NP-like cells effectively and promote the expression of NP-selective ECM-related genes.

### 3.4. Biochemical Content of the Codelivery System

**Figure 7:**
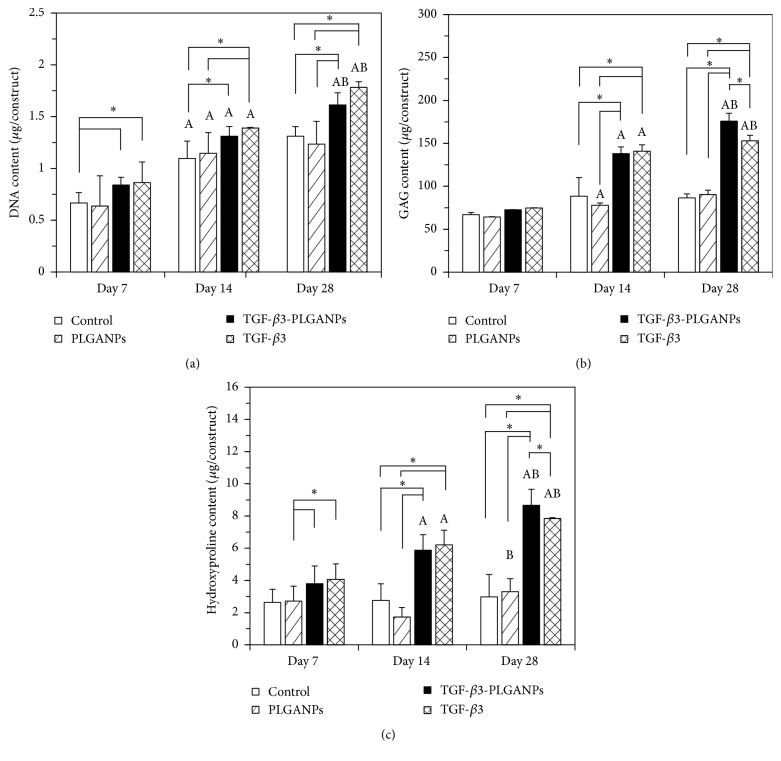
Measurement of the biochemical content of the hybrids including (a) DNA, (b) GAG, and (c) HYP of the MSCs-seeded dextran/gelatin hydrogels encapsulated with no supplement (control), 1000 *μ*g/mL blank PLGANPs (PLGANPs), 1000 *μ*g/mL TGF-*β*3-loaded PLGANPs (TGF-*β*3-PLGANPs), and the hydrogels with no supplements cultured in the medium containing 10 ng/mL TGF-*β*3 (TGF-*β*3) for 7, 14, and 28 days. Data were expressed as means ± SD (*n* = 3). The samples indicated with (*∗*) had a significant difference between the two groups (*p* < 0.05). The samples indicated with (A) had a significant difference compared to the same group on day 7 (*p* < 0.05) and (B) had a significant difference compared to the same group on day 14 (*p* < 0.05).

As shown in Figure 7(a), the results of the DNA content assay agreed with the CCK-8 assay. The enhanced cell proliferation activity was also evidenced by greater elevated DNA content at day 28 than at days 7 and 14 in the hybrid with TGF-*β*3-loaded PLGANPs (1.9- and 1.2-fold, resp., *p* < 0.05).

As shown in Figure 7(b), the GAG content was elevated constantly during the culture period in the hybrid with the TGF-*β*3-PLGANPs (1.9-fold to 2.4-fold higher than it on day 7, *p* < 0.05). The GAG content was significantly greater in the TGF-*β*3-PLGANPs group compared with the control and the PLGANPs group after 14 and 28 days (*p* < 0.05), but not 7 days of postseeding (*p* > 0.05). The GAG content was significantly higher in the TGF-*β*3-PLGANPs group than the TGF-*β*3 group on day 28 (1.2-fold higher, *p* < 0.05).

As shown in Figure 7(c), the HYP content was 1.0- and 1.1-fold greater after 14 and 28 days of culture in the control group, respectively, compared with day 7 (*p* < 0.05). For the TGF-*β*3-PLGANPs group, the HYP content was 1.5- and 2.3-fold greater, respectively, compared with day 7 (*p* < 0.05) after 14 and 28 days of culture. And there is significantly higher HYP content in the TGF-*β*3-PLGANPs groups than the PLGANPs group during the whole period (*p* < 0.05). After 28 days of postseeding, the HYP content was higher significantly in the TGF-*β*3-PLGANPs group than in the TGF-*β*3 groups (1.1-fold, *p* < 0.05).

**Figure 8:**
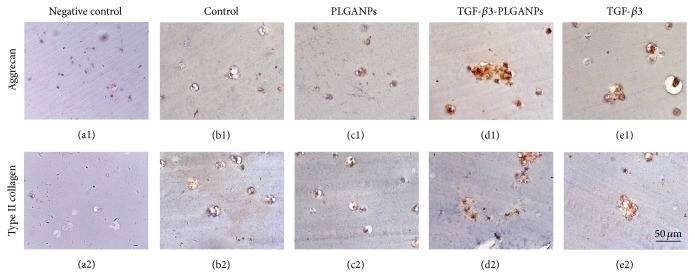
Photomicrographs showed the immunohistological staining for aggrecan (a1–e1) and type II collagen (a2–e2) of neo-NP tissue formation in the codelivery system of the MSCs-seeded dextran/gelatin hydrogel encapsulated with no supplement (control (b1-2)), 1000 *μ*g/mL blank PLGANPs (PLGANPs (c1-2)), 1000 *μ*g/mL TGF-*β*3-loaded PLGANPs (TGF-*β*3-PLGANPs (d1-2)), and the hydrogels with no supplements cultured in the medium containing 10 ng/mL TGF-*β*3 (TGF-*β*3 (e1-2)) for 28 days. (a1-2) were negative controls for immunostaining (magnification: 400x, scale = 50 *μ*m).

As shown in Figure 8, the histological staining showed MSCs in all the groups exhibiting the rounded morphology as the native NPCs. There are more cell clusters in the hybrid with TGF-*β*3-loaded PLGANPs and medium containing TGF-*β*3. Immunohistochemical analysis revealed the ECM distributed in the cytoplasm and the pericellular region. Higher deposition of aggrecan and type II collagen could be found in the TGF-*β*3-PLGANPs and TGF-*β*3 groups. The lowest deposition of ECM could be found in the PLGANPs and the control group. These results indicated the cell proliferation and ECM-related biosynthesis of MSCs could be promoted by the TGF-*β*3-PLGANPs.

## 4. Discussion

Due to the accelerated aging population, therapy for IDD has been widely considered in recent years. Biological therapy for IDD may be better suited to alleviate pain, repair degenerative NP, and restore the biomechanical function of the IVD than traditional treatments [31]. However, appropriate implantation and delivery strategies are still being explored. The advantage of the codelivery system established in the present study is that it could be applied as a minimally invasive injection and may promote long-term differentiation into NP-like cells* in situ*. The purpose of this study was to evaluate the potential of a hydrogel with TGF-*β*3-loaded PLGANPs to induce long-term differentiation of MSCs and promote NP regeneration.

In this codelivery system, the hydrogel described is crosslinked by a Schiff-based reaction. The compositions of dextran and gelatin include natural materials, providing an adhesive site for encapsulated cells [32]. Their biodegradability also provides space for the cell proliferation and ECM secretion. Meanwhile, the Oxi-Dex and amino-Gel prepolymers are in a liquid state while the gelation time was approximately 58 seconds after being mixed, which is feasible for a clinical injectable application. Although the dextran/gelatin hydrogel is mechanically weak as an implant for NP, an implanted scaffold would not carry too much compression in the early stage of IDD, because the mechanical structure is not damaged. Additionally, the aldehyde groups of the material could react with the surface of the tissue, resulting in the hydrogel fixing to the tissue surface through a covalent bond [33]. The tissue integration would prevent the leakage of encapsulated MSCs and PLGANPs and promote the long-term beneficial effect of the codelivery system. Thus, the dextran/gelatin hydrogel is a suitable carrier for MSCs.

The release percentage and time frame of the drug depend on the carriers' degradation rate. PLGA is a well-known carrier for drug delivery systems. In general, the lower molecular weight of PLGA with a higher ratio of PGA is degraded rapidly because of its hydrophilic and amorphous state caused by a higher glycolic acid content. On the contrary, the higher molecular weight of PLGA with a higher ratio of PLA makes nanoparticles more hydrophobic, which slows degradation [34]. Thus, the release time of the drug could be easily controlled from weeks to months by adjusting the PLA/PGA ratio. According to the chondrogenesis process, TGF-*β*3 is required for sustained release for more than one month [35]. Meanwhile, TGF-*β*3 is a hydrophilic protein, similar to PGA. Therefore, PLGA with a relatively low molecular weight and a higher PGA content (PLA : PGA = 50 : 50) was chosen to reach relatively rapid degradation as the carrier material used in this study.

SEM scanning showed that the TGF-*β*3-loaded nanoparticles were spherical with a smooth appearance. As the TEM image showed, there is no obvious adhesion between particles, indicating proper dispersion. It would be beneficial if nanoparticles evenly and uniformly distributed the TGF-*β*3 concentration in the hydrogel. In previous studies, a hydrogel with TGF-*β*-loaded microspheres was constructed and exhibited favorable chondrogenesis activity of MSCs [36, 37]. In contrast with these micron-sized microspheres, the injectability could be significantly improved by using a nanosized PLGA in the codelivery system. Needle puncture can aggravate IDD and is even used to establish an animal model of IDD [38]. Rabbit IVDs subjected to annular needle punctures using 16 to 21 G needles led to degeneration after 8 weeks [38, 39]. Owing to the intact structure of the IVD in the early stage of IDD, the annulus structure should be protected effectively. However, injection could not be avoided when cell-based therapy was applied to early stage IDD. It is encouraging that PLGANPs could be injected with much finer needles than micron-sized microspheres, alleviating annular injury. Therefore, PLGANPs are particularly suitable as drug carriers for the treatment of early stage IDD.

A desired drug delivery rate is one essential criterion for a successful drug delivery system. Generally, the drug release could be roughly divided into the following three stages: the burst release period, the stable period, and the accelerated period. The nanoparticles exhibited a relatively lower burst release on the first day. Then, TGF-*β*3 was stably released from the nanoparticles from days 2 to 14. Cumulative release was approximately 56.4% in the first 28 days. In general, the TGF-*β*3-loaded PLGANPs in this study established a depot with a low burst release, which is ideal for long-term continuous drug release and would meet the requirement of discogenesis differentiation* in situ*. In a previous study, TGF-*β*3 could also be immobilized on the scaffold for controlled release [40]. The drug-immobilized scaffold could release TGF-*β*3 in a long-term culture and induce chondrogenic differentiation. However, the TGF-*β*3 crosslinked on the scaffold usually exhibited a higher burst release. High concentration of TGF-*β*3 above 10 ng/mL may be detrimental to chondrogenesis of MSCs as reported by Clarke et al. [23]. Therefore, the stable release system of PLGANPs in this study is probably a suitable alternative for long-term chondrogenesis.

The cell viability in all of the groups was greater than 94%, indicating that the TGF-*β*3-loaded nanoparticles and blank nanoparticles both exhibited favorable cytocompatibility. To investigate the cell proliferation of the hydrogel with TGF-*β*3-PLGANPs during the period of MSC discogenesis, the proliferation of encapsulated MSCs was assessed for 28 days. As shown in Figure 4, the PLGANPs alone had no obvious impact on cell proliferation during the testing period. In the TGF-*β*3-PLGANPs and TGF-*β*3 groups, the cell proliferation of MSCs was enhanced significantly, which has been described by Shi and Massagué [41]. The phenomenon could be attributed to the fact that TGF-*β*3 could activate the Smad complex and subsequently activate the p15Ink4b promoter [42]. This could also be evidenced by the gradually increasing DNA content in the codelivery system. The results demonstrated that the high metabolite activity of MSCs was maintained in the hydrogel with TGF-*β*3-loaded nanoparticles, laying a foundation for the differentiation.

Due to the low cell density present in the NP, transplanting a large number of autologous cells, such as MSCs, with an appropriate carrier may be an effective strategy for promoting new tissue formation [43]. For this strategy, a properly differentiated phenotype is the basis of the regeneration of the NP. MSCs could differentiate toward a chondrogenic lineage with the assistance of TGF-*β*3, which is proven by the upregulation of aggrecan (ACAN) and type II collagen (Col2) *α*1. However, due to a lack of NPC phenotypes, native NPCs are always treated as chondrocyte-like cells and identified by the expression of chondrogenesis markers, such as SOX-9, Col2, and ACAN. Thus, it could not be determined whether MSCs actually differentiated into NPCs in many studies. However, the composition and biological function of chondrocytes and NPCs are obviously different [44]. The ECM secreted by chondrocytes is much stiffer than that released by NPCs and is not suitable for fluid pressurization in the NP. Thus, improper differentiation may result in a failure to restore IVD function. If MSCs are chosen as the cell source to promote NP regeneration, it is necessary to identify the cell phenotype of differentiated cells to ensure proper ECM deposits [45]. Some markers identified in rat arrays showed relatively significant differential expression in human NPCs versus articular cartilage cells, especially KRT18 [46]. These results were also confirmed at the protein level with immunohistochemistry [47]. Additionally, sonic hedgehog (Shh) is a ligand of the hedgehog family, and it was selectively expressed in young NP, as Dahia et al. reported [48]. Shh is required for the proper functioning of NPCs and is a potential vital phenotype of NPCs [49]. Encapsulating MSCs in a hydrogel with TGF-*β*3-PLGANPs led to a significant increase in the NP phenotypic marker genes of KRT18 and Shh relative to the control and PLGANP groups. In particular, there was a much higher expression of Shh in the TGF-*β*3-PLGANP group than in the TGF-*β*3 group. These positive results indicated that the codelivery system could efficiently induce the discogenesis differentiation of MSCs triggered by TGF-*β*3* in situ*, establishing a foundation for enhanced biosynthesis of implanted cells.

The biosynthetic activity of MSCs in the presence of TGF-*β*3-loaded nanoparticles was evidenced by increasing GAG and HYP with an extended culture time. Interestingly, proteoglycan synthesis was slightly higher in the hydrogel with TGF-*β*3-loaded PLGANPs than in the TGF-*β*3-supplemented culture medium on day 28. There are two potential explanations for this phenomenon. On the one hand, due to the bulk size of the hydrogel, the TGF-*β*3 in the medium may be unable to penetrate into the central region of the hydrogel. The concentration gradient of TGF-*β*3 could decline from the outer to the inner area. On the contrary, the nanoparticles could distribute uniformly in the hydrogel as mentioned above. Thus, TGF-*β*3 could reach the inner region of the hydrogel and activate more MSCs in the hydrogel. On the other hand, the half-life of TGF-*β*3 in the medium was relatively short. With the extension of the culture time, the biological effects observed with the shorter duration of TGF-*β*3 were gradually increased.

Clusters of MSCs in a chondrocyte-like rounded shape in the codelivery system indicated cells were in a state of proliferation in the area of the degraded scaffold and were moving toward discogenesis differentiation. The findings also indicated that aggrecan and type II collagen expression were upregulated in the TGF-*β*3-PLGANP and TGF-*β*3 groups. The ECM-related proteins started to distribute in the pericellular region, indicating new NP tissue formation. Due to the relatively short culture period, the scaffold was not degraded enough to provide more space for tissue formation. Further experiments would need to be conducted to determine the effect of long-term culture.

In the present study, a limitation was that the hybrid was cultivated* in vitro*, where the medium condition is suitable for MSC proliferation. This may have influenced MSC viability and affected the long-term regeneration in the harsh microenvironment of the NP* in vivo*, which is of high acidity and low nutrient supply [50, 51]. In addition, we found that the structure of the hydrogel at the concentration of 10% was easily damaged following long-term culture* in vitro* in our previous study. Thus, we applied the hydrogel with a higher concentration of 20% and found that the structure was stable enough, but this may compromise its injectable property. In the future, an* in vivo* study should be conducted to explore the viability and biosynthetic situation of MSCs and test the feasibility of the codelivery system for NP injection.

## 5. Conclusion

In summary, in this series of* in vitro* studies, we successfully established a codelivery system of a dextran/gelatin hydrogel with TGF-*β*3-loaded PLGANPs. The nanoparticles could stably release active TGF-*β*3 for long term. The differentiating potential of this novel hybrid was validated by inducing MSCs into discogenesis cells* in situ* during the period of 28 days. It is possible that the codelivery system may be applied for the treatment of early stage IDD using autologous MSCs. In future studies, we will investigate the long-term regenerative effect of MSCs using the delivery hybrid in a large-animal model of IDD.

## Figures and Tables

**Table 1:** Real-time polymerase chain reaction primers.

Gene	Sequence	Size
Aggrecan	Forward: 5′ ATTTCCACACGCTACACCCTG 3′	164 bp
	Reverse: 5′ TGGATGGGGTATCTGACTGTC 3′	

Type II collagen *α*1	Forward: 5′ CAGGATGCCCGAAAATTAGGG 3′	132 bp
	Reverse: 5′ ACCACGATCACCTCTGGGT 3′	

KRT18	Forward: 5′ TCCATCAGGGTGACTCAGAAA 3′	250 bp
	Reverse: 5′ CCAGCTTCAAGGGGCTCAA 3′	

Shh	Forward: 5′ AAAGCTGACCCCTTTAGCCTA 3′	123 bp
	Reverse: 5′ TTCGGAGTTTCTTGTGATCTTCC 3′	

GAPDH	Forward: 5′ GGAGTTGCTGTTGAAGTCGCA 3′	532 bp
	Reverse: 5′ GGAGTTGCTGTTGAAGTCGCA 3′	

Primers for aggrecan, type II collagen *α*1, Shh, and GAPDH were designed from mus musculus gene sequences obtained from the NCBI GenBank and RefSeq databases using primer 5.0 software.
